# „Resuscitative endovascular balloon occlusion of the aorta“ (REBOA): Aktuelles zu Material, Indikationen und Grenzen: ein Überblick

**DOI:** 10.1007/s00104-020-01180-0

**Published:** 2020-06-08

**Authors:** M. Wortmann, M. Engelhart, K. Elias, E. Popp, S. Zerwes, Alexander Hyhlik-Dürr

**Affiliations:** 1 Klinik für Gefäßchirurgie und Endovaskuläre Chirurgie, Universitätsklinikum Heidelberg, Heidelberg, Deutschland; 2 Klinik für Gefäßchirurgie und Endovasku­läre Chirurgie, Bundeswehrkrankenhaus Ulm, Ulm, Deutschland; 3 Abteilung für Gefäßchirurgie, Bundeswehrkrankenhaus Westerstede/Ammerland Klinik, Westerstede, Deutschland; 4 Sektion Notfallmedizin, Klinik für Anästhesiologie, Universitätsklinikum Heidelberg, Heidelberg, Deutschland; 5 Gefäßchirurgie und endovaskuläre Chirurgie, Medizinische Fakultät, Universitätsklinikum Augsburg, Stenglinstr. 2, 86156 Augsburg, Deutschland

**Keywords:** REBOA, Ballon Blockade Aorta, REBOA Technik, REBOA Komplikationen, Polytrauma, REBOA, Ballon aortic occlusion, REBOA technique, REBOA complications, Polytrauma

## Abstract

**Hintergrund:**

„Resuscitative endovascular balloon occlusion of the aorta“ (REBOA) stellt ein endovaskuläres Verfahren dar, bei dem ein Blockballon in die Aorta eingeführt wird, um eine distal des Ballons gelegene Blutung zu verringern und gleichzeitig die kardiale und zerebrale Oxygenierung zu verbessern.

**Ziel der Arbeit:**

Vorstellung der REBOA-Technik, der möglichen Indikationen, der benötigen Materialien und der möglichen Komplikationen des Verfahrens.

**Material und Methoden:**

Nichtsystematischer Übersichtsartikel über die aktuelle Literatur.

**Ergebnisse:**

REBOA stellt gerade bei traumatisch bedingten Blutungen und rupturierten Aortenaneurysmen ein mögliches additives Verfahren zur hämodynamischen Stabilisierung dar. Die Komplikationsrate des Verfahrens liegt bei ungefähr 5 %, wobei Zugangskomplikationen im Vordergrund stehen, jedoch auch letale Komplikationen möglich sind.

**Diskussion:**

Eine aortale Ballonblockade wird bei der Versorgung rupturierter Aortenaneurysmen standardmäßig eingesetzt. Es gibt wachsende Evidenz, dass REBOA bei der Versorgung polytraumatisierter Patienten mit einem hämorrhagischen Schock aufgrund einer abdominellen oder viszeralen Blutung eine vergleichsweise minimal-invasive Alternative zur offen chirurgischen Aortenklemmung mittels Thorakotomie darstellt. Mit der Entwicklung neuer Ballonkatheter, die ohne Führungsdraht und mit geringeren Schleusendurchmessern auskommen, wird auch ein Einsatz bei anderen Krankheitsbildern wie postoperativen abdominellen Nachblutungen, gynäkologischen Blutungen oder als additives Verfahren bei der kardiopulmonalen Reanimation diskutiert.

## Abstract (en)

**Background:**

Resuscitative endovascular balloon occlusion of the aorta (REBOA) describes an endovascular procedure in which a blocking balloon is introduced into the aorta to reduce bleeding situated distal to the balloon and simultaneously to improve cardiac and cerebral oxygenation.

**Objective:**

Presentation of the REBOA technique, the possible indications, the required material and possible complications of the procedure.

**Material and methods:**

Non-systematic review of the currently available literature.

**Results:**

The REBOA procedure is an adjunct to achieve hemodynamic stabilization in patients with traumatic hemorrhage and ruptured aortic aneurysms. The complication rate of the procedure is approximately 5%, whereby access complications are the most common; however, fatal complications are also possible.

**Conclusion:**

A balloon block of the aorta is well established in the treatment of ruptured aortic aneurysms. There is growing evidence that REBOA is a minimally invasive alternative to open surgical cross-clamping of the aorta by thoracotomy for the treatment of patients with polytrauma and hemorrhagic shock due to abdominal or visceral bleeding. Due to the development of new balloon catheters, which can be placed without stiff guidewires and require smaller sheath diameters, REBOA is also discussed for treatment of postoperative abdominal or gynecological bleeding or as a possible adjunct to cardiopulmonary resuscitation for nontraumatic cardiac arrest.

„Resuscitative endovascular balloon occlusion of the aorta“ (REBOA) ist ein endovaskuläres Verfahren, bei dem ein Blockballon über einen Leistenzugang in der Aorta platziert und inflatiert wird. Damit soll bei traumatischen und nichttraumatischen Blutungen im Bereich des Abdomens und des Beckens eine Reduktion des Blutverlustes in Kombination mit einer Verbesserung der zerebralen und koronaren Durchblutung erreicht werden. Somit stellt REBOA eine endovaskuläre Alternative zur offen chirurgischen Aortenklemmung mittels Thorakotomie für Patienten in extremis dar.

Der Blockballon kann in Abhängigkeit von der Indikation respektive der vermuteten Lokalisation der Blutung entweder in der infrarenalen Aorta (REBOA-Zone-III) oder in der Aorta descendens (REBOA-Zone-I) platziert werde (Abb. 1).

**Figure 1:**
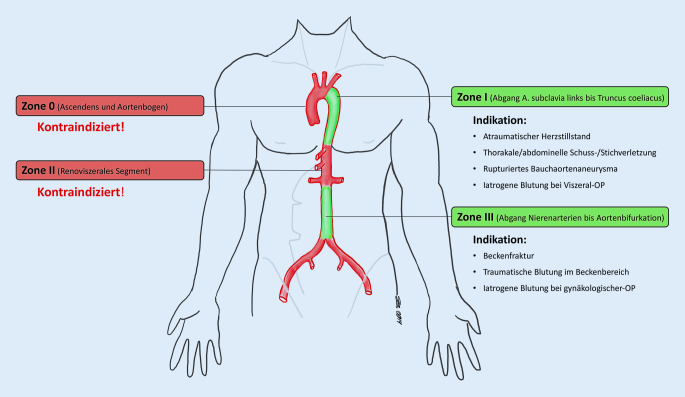

REBOA kam bereits während des Korea-Krieges in den 1950er-Jahren zum Einsatz, ist aber seitdem wieder in Vergessenheit geraten [1]. Durch die zunehmende Verbreitung endovaskulärer Techniken in der Versorgung polytraumatisierter Patienten erlebt das Verfahren momentan jedoch eine Renaissance und stellt zurzeit ein diskutiertes Thema in der Notfallmedizin dar. Dies wird nicht nur durch vermehrte Sitzungen zu REBOA auf traumatologischen und notfallmedizinischen Fachtagungen deutlich. Inzwischen gibt es sogar internationale Kongresse, die sich nur mit der Anwendung endovaskulärer Verfahren bei der Traumaversorgung beschäftigen (z. B. Endovascular Resuscitation and Trauma Management (EVTM) Symposium). Auch die rapide steigende Anzahl von mehr als 130 PubMed gelisteten Publikationen allein in den Jahren 2018 und 2019 zeigt das Interesse an dieser wiederentdeckten Technik.

Gerade bei traumatologischen Patienten mit einem hämorrhagischen Schock aufgrund einer Blutung des Abdomens oder des Beckens scheint der Einsatz eines Ballons zur Okklusion der Aorta zur Blutungskontrolle und Aufrechterhaltung der lebensnotwendigen zerebralen und kardialen Perfusion ein vielversprechendes Verfahren zur Verbesserung der Überlebensraten und der Behandlungsergebnisse zu sein. Zudem stellt es gerade bei Patienten in extremis im Vergleich zur offen chirurgischen Aortenklemmung, die bei traumatologischen Patienten nur mit einer Überlebensrate von 10 % einhergeht [2, 3], einen Eingriff mit bedeutend geringerer Invasivität dar.

Über die Anwendung von REBOA bei traumatologischen Blutungen des Körperstammes und stammnahen Blutungen hinaus wird die endovaskuläre Ballonblockade der Aorta auch bei nichttraumatologische Hämorrhagien wie z. B. bei gynäkologischen Blutungen, gastrointestinalen Blutungen und postoperativen Nachblutungen nach abdominellen Eingriffen diskutiert [4, 5]. Nicht zuletzt ist auch eine Anwendung im Rahmen nichttraumatisch bedingter Herz-Kreislauf-Stillstände denkbar.

Durch dieses erweiterte Indikationsspektrum ist REBOA inzwischen nicht nur für Notfallmediziner und Traumatologen von Interesse. Die Technik, das Indikationsspektrum, aber auch Gefahren durch die Anwendung sollten allen chirurgisch tätigen Kolleginnen und Kollegen geläufig sein.

Im folgenden Übersichtsartikel soll die Technik von REBOA einschließlich der verfügbaren und benötigten Materialien vorgestellt werden, die möglichen Einsatzgebiete aufgezeigt, aber auch mögliche Komplikationen durch den Einsatz von REBOA beschrieben werden, um eine neutrale Beurteilung der Methode zu ermöglichen.

## Indikationen

### Traumatisch bedingte Blutungen

REBOA stellt eine weniger invasive, endovaskuläre Alternative zur Notfallthorakotomie mit supradiaphragmaler Klemmung der Aorta bei nichtkomprimierbaren Blutungen im Bauch- und Beckenraum dar. Während tierexperimentelle Studien die Überlegenheit der Ballonokklusion mit zuverlässigem Anstieg des zentralen Perfusionsdrucks bei gleichzeitig geringerem physiologischem Stress belegen konnten [6], ist die Evidenz hinsichtlich eines besseren Behandlungsergebnisses bei Traumapatienten nach wie vor schwach. Dies liegt zum einen an der hohen Letalität der meist polytraumatisierten Patienten, zum anderen an dem selbst in großen Traumazentren kleinen Fallzahlen mit großer Heterogenität des Verletzungsmusters.

Ersten vielversprechenden Fallserien aus dem zivilen [7] und militärischen Bereich [8] zu REBOA bei Trauma folgten widersprüchliche größere Serien. Erste Auswertungen des „Aortic Occlusion for Resuscitation in Trauma and Acute Care Surgery-Registry“, in dem Daten zu REBOA und zur Notfallthorakotomie aus großen US-Traumazentren verglichen werden, belegen eine Überlegenheit von REBOA [9, 10]. Die jüngste Publikation dieser Daten umfasste 285 Patienten (Notfallthorakotomie 71 % vs. REBOA 29 %) und zeigt bei vergleichbarer Verletzungsschwere einen signifikanten Überlebensvorteil für die REBOA-Gruppe (gesamt Überlebensrate: Notfallthorakotomie 44 % vs. REBOA 63 %), vor allem bei Patienten ohne kardiopulmonaler Reanimation (Überlebensrate bis Entlassung: Notfallthorakotomie 0 % vs. REBOA 44 %; [10]). Kritiker bemängeln jedoch den direkten Vergleich beider Verfahren, da REBOA und Notfallthorakotomie nicht notwendigerweise die gleichen Indikationen hätten [11]. In einer retrospektiven Fall-Kontroll-Studie, bei der 140 Patienten, die REBOA erhalten hatten, 280 Patienten ohne REBOA als Vergleichsgruppe zugeordnet wurden, war kein Unterschied in Bezug auf die Menge der verabreichten Blutprodukte oder die Länge des Krankenhausaufenthaltes nachweisbar. Allerdings fanden Joseph et al. eine höhere Mortalität in der REBOA-Gruppe (36 % vs. 19 %), eine höhere Rate an akutem Nierenversagen (11 % vs. 3 %) und Amputationen der unteren Extremitäten (4 % vs. 1 %; [11]). Die Autoren führten diese Unterlegenheit auf den Zeitverlust bis zur Operation und die viszerorenale Ischämiezeit in der REBOA-Gruppe zurück.

In einer Metaanalyse zeigte sich ein signifikanter Überlebensvorteil bei traumatologischen Patienten durch die Verwendung von REBOA [12]. Bei polytraumatisierten Patienten mit hämorrhagischem Schock konnte durch den Einsatz von REBOA der zentrale systolische Blutdruck um knapp 80 mmHg angehoben werden, was die Effektivität des Verfahrens unterstreicht. Die Komplikationsrate durch das Verfahren lag in dieser Studie bei 5 %.

Zusammenfassend gibt es also noch wenig Evidenz bezüglich der Überlegenheit von REBOA im Rahmen der Versorgung polytraumatisierter Patienten, wobei die bislang publizierten Daten einen guten Effekt in Bezug auf die hämodynamische Stabilisierung bei akzeptabler Komplikationsrate zeigen. Somit erscheint REBOA durchaus als valide Alternative zur Notfallthorakotomie. Da jedoch auch negative Daten bezüglich des Behandlungsergebnisses vorliegen, sind weitere Studien notwendig, um die Indikationsstellung zu verbessern und somit Patienten zu identifizieren, die von dem Verfahren profitieren können.

In der aktuellen S3-Leitlinie Polytrauma/Schwerverletztenbehandlung in der aktualisierten Fassung von 2016 wird REBOA als mögliche Maßnahme zur Behandlung kreislaufinstabiler Patienten in extremis aufgeführt, allerdings mit niedrigem Empfehlungsgrad [13].

Dies deckt sich mit internationalen Empfehlungen, z. B. des American College of Surgeons Committee on Trauma (ACS COT) und des American College of Emergency Physicians (ACEP; [14]).

Basierend auf den Daten des TARN (Trauma Audit and Research Network) in England und Wales errechneten Barnard et al. eine Rate von 4 potenziellen REBOA-Patienten pro Jahr in Major Trauma Centres (MTC) und von 9 Patienten pro Jahr in den größten 10 Traumazentren [15]. In kleineren Trauma-Units ist statistisch sogar nur alle 3 Jahre mit einem REBOA-Patienten zu rechnen. In einer retrospektiven Analyse des TraumaRegister DGU® zeigten sich für Deutschland ähnlich niedrige Raten von einem Patienten pro Jahr in überregionalen, 0,1 Patienten pro Jahr in regionalen und 0,01 Patienten pro Jahr in lokalen Traumazentren [16]. Diese niedrigen Inzidenzen wiederum stellen die Implementierung von REBOA inklusive der Vorhaltung des notwendigen Materials und die Schulung des Personals infrage, falls diese Strukturen nicht sowieso bereits lokal vorgehalten werden, z. B. für die Versorgung rupturierter Aortenaneurysmen.

### Rupturiertes Aortenaneurysma

Bei der Versorgung rupturierter Aortenaneurysmen ist eine endovaskuläre Ballonblockade im Sinne eines REBOA-Manövers ein etablierter Teil der Versorgungsstrategie bei hämodynamisch instabilen Patienten [17]. Gerade bei der endovaskulären Versorgung bietet sich das Einbringen eines Blockballons über die in den Leisten einliegenden Schleusen an. Durch einen intraoperativen Wechsel des Blockballons kann eine komplette endovaskuläre Aneurysmaausschaltung unter Ballonblockade der Aorta erfolgen [18]. Aber auch bei der offen chirurgischen Therapie eines rupturierten Aortenaneurysmas ist die Platzierung eines aortalen Blockballons ein probates Mittel zur hämodynamischen Stabilisierung des Patienten bis zur offen chirurgischen Aortenklemmung. Da in Aortenzentren das für das Manöver benötigte Material und die Expertise vorhanden sind, bietet sich hier auch eine breitere Anwendung des REBOA-Manövers im Rahmen anderer Akutsituationen an.

### Peripartale gynäkologische Blutungen

Auch peripartale gynäkologische Blutungen, z. B. bei Placente previa, stellen eine mögliche Indikation für REBOA dar. Hierbei könnte nicht nur der Blutverlust während der Operation reduziert werden, sondern auch die Rate an Hysterektomien verringert werden. Einschränkend muss erwähnt werden, dass für diese Indikation die Datenlage noch geringer ist als für die Anwendung von REBOA bei traumatologischen Patienten oder im Rahmen der Versorgung rupturierter Aortenaneurysmen. In einer exemplarischen Fallserie von 15 Patientinnen, die im Rahmen der geplanten Sectio bei Placenta previa REBOA erhalten hatten, war die Notwendigkeit zur Hysterektomie und die Menge an transfundierten Blutprodukten im Vergleich zur Kontrollgruppe deutlich reduziert [19]. In dem bereits erwähnten systematischen Review aus dem Jahr 2018 sind bereits 5 Einzelfallbericht respektive kleinere Fallserien eingeschlossen [12]. Gerade bei einer geplanten Sectio mit erwartetem größerem Blutverlust scheint die früh elektive Anlage einer arteriellen Schleuse in der Leiste unter duplexsonographischer Kontrolle vor Beginn des Eingriffs ein probates Mittel, um im Falle einer während der Operation eintretenden starken Blutung direkt einen aortalen Blockballon in die infrarenale Aorta vorbringen zu können. Dabei erscheinen vor allem spezielle REBOA-Katheter mit geringer Schleusengröße vielversprechend, da hierdurch das Zugangstrauma minimiert wird (siehe Technik und Material).

### Nichttraumatische abdominelle oder pelvine Blutungen

Auch nichttraumatische Blutungen im Bereich des Abdomens oder Beckens können eine Indikation für REBOA darstellen. Hierzu liegen ebenfalls bereits verschiedene Einzelfallberichte sowie kleinere Fallserien vor, die den Einsatz einer aortalen Ballonblockade bei oberen gastrointestinalen Blutungen [20], rupturierten Viszeralarterienaneurysmata [21] oder Nachblutungen im Rahmen viszeralchirurgischer Operationen [22], insbesondere Pankreasoperationen [23], beschreiben. Vergleichbar mit der Verwendung bei traumatisch bedingten Blutungen bewirkt REBOA bei dieser Form der Blutungen im Durchschnitt einen Blutdruckanstieg von ca. 50 mm Hg [24].

### Nichttraumatischer Herz-Kreislauf-Stillstand

Auch beim nichttraumatischen Herz-Kreislauf-Stillstand ohne Vorliegen einer Blutung kann durch REBOA eine Zentralisierung des Kreislaufes und somit eine Verbesserung der kardialen und zerebralen Perfusion und Oxygenierung während der Reanimation erreicht werden. Somit wird auch in diesem Kontext der Einsatz von REBOA diskutiert, ohne dass diesbezüglich außerhalb von Einzelfallberichten und Fallserien belastbare Evidenz vorliegt [25, 26].

## Technik und Material

Die Idee der aortalen Ballonokklusion bei schwerer traumatischer Blutung wurde bereits in den 50er-Jahren des letzten Jahrhunderts von Colonel Hughes vorgestellt [1]. Wegen der unbefriedigenden Ergebnisse wurde das Verfahren jedoch zunächst nicht weiterverfolgt bis Fortschritte in der endovaskulären Technik und verbesserte Materialien den Einsatz der Ballonokklusion zunächst beim rupturierten Bauchaortenaneurysma rechtfertigten. Mit REBOA im engeren Sinne wurden die Indikationen auf traumatische und zunehmend auch nichttraumatische Blutungen in Abdomen und Becken erweitert.

Zunächst kamen aortale Okklusionsballons, wie sie aus der Gefäßchirurgie bekannt waren, für REBOA zum Einsatz. Weit verbreitet waren der Coda Balloon® (Fa. Cook Medical, USA) und der Reliant Balloon® (Fa. Medtronic, USA; Tab. 1). Beide mussten über einen Führungsdraht unter Durchleuchtung vorgeschoben werden und benötigten eine lange 60 cm (Aorta descendens, REBOA-Zone_I) oder kurze 45 cm (infrarenale Aorta, REBOA-Zone-III) 14-Fr- bzw. 12-Fr-Schleuse zur Stabilisierung in der Aorta. Neben den praktischen Problemen mit Platzierung der 7‑Fr-Einführschleuse in Seldinger-Technik, Wechsel auf eine 12- bis 14-Fr-Schleuse und der Handhabung langer, steifer Führungsdrähte, welche für den nichtendovaskulär geübten Traumachirurgen eine ungewohnte Herausforderung darstellten und Zeit kosteten, provozierten vor allem die kaliberstarken Schleusen Komplikationen wie großes Zugangstrauma in der Leistenarterie mit Nachblutungen und Pseudoaneurysmata sowie Thrombosen in den ipsilateralen Becken- und Beinarterien bis hin zur sekundären Majoramputation bei akuter Extremitätenischämie [27].

**Table 1:** 

Produkt	Ballondurchmesser	Schleusengröße	Führungsdraht für Katheter	Operativer Gefäßverschluss
Coda® Balloon (Cook Medical, Boomington, USA)	Max. 32 mm	12 Fr 45–60 cm	Ja	Ja
Reliant™ (Medtronic, Minneapolis, USA)	10–46 mm	12 Fr 45–60 cm	Ja	Ja
Rescue Balloon® (Tokai Medical, Sarayashiki Taraga Kasugai-city, Japan)	16–40 mm	7 Fr	Ja	Nein
ER-REBOA™ (Prytime Medical, Boerne TX, USA)	–	7 Fr	Nein	Nein
Reboa Balloon Kit™ (Reboa Medical AS, Norway)	15–30 mm (Zone III) 20–30 mm (Zone I)	6/7 Fr 8–15 cm	Ja	Nein

**Table 2:** 

Komplikation	Autor, Jahr	Rate	
*Gefäßassoziierte Komplikationen*			
Aortoiliakale Verletzung (z. B. Intimaläsion, Dissektion, Thrombose, Ruptur)	Pieper et al., 2018 [32]	–	
	Brenner et al., 2018 [14]		
	Morrison et al., 2016 [33]		
	Davidson et al., 2018 [34]		
Pseudoaneurysma	Sadek et al., 2016 [35]	2,2–6,5 % DuBose	
	Conti et al., 2017 [36]		
	DuBose et al., 2016 [9]		
Thrombembolie, Ischämie	DuBose et al., 2016 [9]	0,5–14,2 % Morrison, DuBose, Saito, Sadeghi	
	Pieper et al., 2018 [32]		
	Davidson et al., 2018 [34]		
	Gamberini et al., 2017 [37]		
	Martinelli et al., 2010 [38]		
	Morrison et al., 2016 [33]		
	Saito et al., 2015 [39]		
	Sadeghi et al., 2018 [40]		
Majoramputation	Joseph et al., 2019 [11]	3,6–21,3 % Joseph, Saito	
	Saito et al., 2015 [39]		
	Sadek et al., 2016 [35]		
	Andres et al., 2016 [41]		
	Gamberini et al., 2017 [37]		
*Technische Probleme*			
Ballonmigration	DuBose et al., 2016 [9]	0,2–4 % DuBose, Sadeghi	0,8 % (Mortalität) Morrison
	Sadeghi et al., 2018 [40]		
Ballonruptur	Hörer, 2016 [42]	0,1–3 % Martinelli, Sadeghi	
	Martinelli et al., 2010 [38]		
	Brenner et al., 2018 [14]		
	Sadeghi et al., 2018 [40]		
Einführung, Katheterplatzierung	Davidson et al., 2018 [34]	–	
	Gamberini et al., 2017 [37]		
*Sonstige Komplikationen*			
Akutes Nierenversagen	Joseph et al., 2019 [11]	10,7–35,7 % Saito, Joseph, Pieper	
	Pieper et al., 2018 [32]		
	Morrison et al., 2016 [33]		
	Saito et al., 2015 [39]		
	Conti et al., 2017 [36]		
	Davidson et al., 2018 [34]		
Spinale Ischämie	Brenner et al., 2018 [14]	–	
Zerebrale Blutung	Gamberini et al., 2017 [37]	0,1 % Gamberini	
	Uchino et al., 2016 [43]		
Schwere Rhabdomyolyse	Pieper et al., 2018 [32]	46,9 % Pieper	
Kompartmentsyndrom	Wasicek et al., 2018 [44]	0,7–15 % Joseph, Sadeghi, Wasicek	
	Sadeghi et al., 2018 [40]		
	Joseph et al., 2019 [11]		

Neue Entwicklungen spezieller Ballonsysteme für REBOA konzentrierten sich daher auf eine Vereinfachung der Handhabung und einen Verzicht auf kaliberstarke Schleusen. Durch den Verzicht auf eine größere Schleuse werden hierbei nicht nur die Komplikationen, sondern auch die Zeit bis zur Inflation des Ballons in der Aorta deutlich reduziert [28]. Der Rescue Balloon® (Tokai Medical, Japan) kommt mit einer kurzen (8–15 cm) 7‑Fr-Einführschleuse aus und stabilisiert sich selbst in der Aorta. Die Platzierung erfolgt jedoch nach wie vor unter Durchleuchtung über einen Führungsdraht. Das Gleiche gilt für den relativ neuen Ballon eines norwegischen Herstellers (Reboa Medical AS, Norwegen), welcher in einem praktischen Reboa Balloon Kit™ einschließlich aller benötigten Materialen geliefert wird.

**Figure 2:**
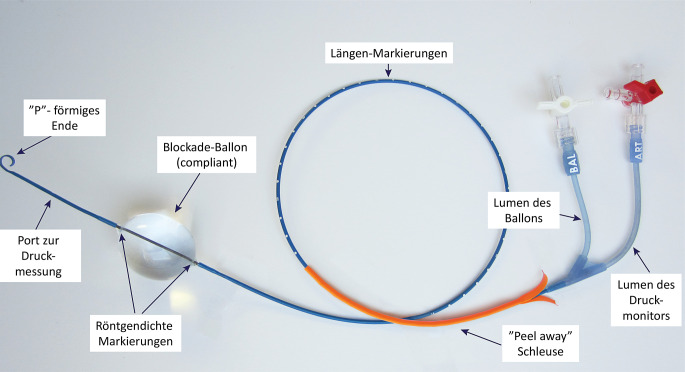

Gänzlich ohne Führungsdraht für den Katheter und notfalls auch ohne Durchleuchtung kommt der ER-REBOA™ (Prytime Medical, USA) aus (Abb. 2). Dieser Ballon wurde in enger Zusammenarbeit mit den Gefäßchirurgen des US-Militärs entwickelt und soll den sicheren und einfachen Einsatz auch unter ungünstigen, prähospitalen Bedingungen erlauben. Nach Abmessung der notwendigen Katheterlänge mithilfe der Markierungen auf dem Schaft wird der Katheter über eine 7‑Fr-Einführschleuse notfalls „blind“ vorgeschoben. Eine atraumatische, abgerundete Spitze (P-tip®), ähnlich der Konfiguration eines Pigtail-Katheters, soll hierbei iatrogene Gefäßverletzung und ein Ausweichen in Seitenäste vermeiden. Ein Kanal mit seitlicher Öffnung erlaubt die Druckmessung und Medikamentenapplikation proximal des insufflierten Ballons. Wie die anderen 7‑Fr-Ballons auch ist der Katheter steif genug, um sich selbst in der Aorta zu stabilisieren. In einem Bauchaortenaneurysma können sich die Katheter jedoch nicht an der Wand abstützen und können somit dislozieren. Wie beim Reboa Balloon Kit™ wird auch für den ER-REBOA™ ein praktisches Komplettset mit allen Zubehörteilen angeboten. Mit Ausnahme des japanischen Rescue Balloon® sind alle vorgestellten Katheter inzwischen auch für den deutschen Markt zugelassen.

Aktuelle Publikationen zeigen, dass sich die Low-profile-REBOA-Ballons gegenüber den kaliberstarken Kathetern weitgehend durchgesetzt haben [27]. Im militärischen Bereich ist der ER-REBOA™ wegen seiner vergleichsweisen einfachen Handhabung weit verbreitet. Weitere Entwicklungen gehen z. B. in Richtung einer Erleichterung der Platzierung des Ballons ohne Durchleuchtung [29].

## Komplikationen

**Figure 3:**
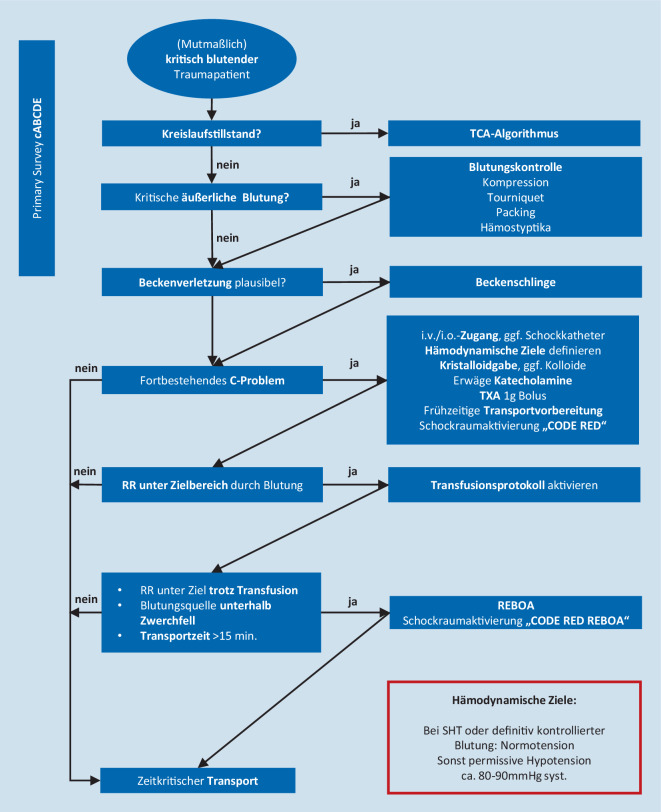

Wie jedes endovaskuläre Verfahren birgt REBOA multiple Komplikationsrisiken. Besonders der perkutane Zugang zur A. femoralis communis im Bereich der Leiste ist bei Patienten unter Reanimation oder minimalem Kreislauf oftmals nicht sicher möglich, sodass Zentren mit größeren Fallzahlen eine relevante Anzahl offen chirurgischer Leistenfreilegungen beschrieben, um überhaupt einen Zugang zum Gefäß zu erlangen. Prinzipiell können die Komplikationen in Komplikationen durch die Malperfusion distal des inflatierten Blockballons, in Zugangskomplikationen sowie Komplikationen durch Dislokation des Drahtes und des Blockballons eingeteilt werden [30]. In dem bereits mehrfach zitierten systematischen Review liegt die Komplikationsrate bei ca. 5 % [12]. Dabei sind besonders bei langer Lagezeit der Blockballons arterielle Thrombosen besonders häufig [31]. Somit ist im Rahmen des Verfahrens eine gefäßchirurgische Expertise vor Ort unbedingt notwendig. Erste Ansätze, REBOA nicht nur im klinischen Setting, sondern auch prähospital einzusetzen, bedürfen einer speziellen Ausstattung der Notfallteams sowie einer standardisierten Patientenselektion, wie dies im Rahmen eines Forschungsprojekts für invasive Notfalltechniken schon etabliert wird (Abb. 3). Eine Zusammenfassung möglicher Gefäßassoziierter Komplikationen zeigt Tab. 2.

## Fazit für die Praxis

- REBOA bezeichnet die endovaskuläre Ballonblockade der Aorta zur hämodynamischen Stabilisierung bei hämorrhagischem Schock.
- REBOA ist ein etabliertes Verfahren bei der Therapie rupturierter Aortenaneurysmen.
- Die aktuelle Evidenz erlaubt den Einsatz von REBOA bei Polytraumatisierten mit einem hämorrhagischen Schock aufgrund einer abdominellen, pelvinen oder stammnahen Blutung als Alternative zur Notfallthorakotomie.
- Neue, speziell für das Verfahren entwickelte Ballonkatheter kommen mit geringeren Schleusendurchmessern aus oder sind durch den Verzicht auf einen steifen Führungsdraht schneller platzierbar.
- Die Komplikationsrate liegt bei ca. 5 %, wobei Zugangskomplikationen im Vordergrund stehen.
- Der Einsatz von REBOA wird auch bei anderen Krankheitsbildern wie gynäkologischen oder postoperativen abdominellen oder pelvinen Nachblutung diskutiert.
- Da auch bereits Komplikationen mit letalen Ausgängen und Fallserien mit einem schlechteren Behandlungsergebnis in der REBOA-Gruppe publiziert sind, sind weitere Studien zur Verbesserung der Patientenauswahl und Indikationsstellung notwendig.
